# Patterning of individual variability in neurocognitive health among South African women exposed to childhood maltreatment

**DOI:** 10.1038/s41598-021-85979-9

**Published:** 2021-03-23

**Authors:** Christy A. Denckla, Sun Yeop Lee, Rockli Kim, Georgina Spies, Jennifer J. Vasterling, S. V. Subramanian, Soraya Seedat

**Affiliations:** 1 Department of Social and Behavioral Sciences, Harvard T. H. Chan School of Public Health, 677 Huntington Avenue, Boston, MA 02115 USA; 2 Interdisciplinary Program in Precision Public Health, Department of Public Health Sciences, Graduate School of Korea University, Seoul, Republic of Korea; 3 Division of Health Policy and Management, College of Health Science, Korea University, Seoul, Republic of Korea; 4 NRF/DST South African Research Chairs Initiative, PTSD Program, Stellenbosch University, Cape Town, South Africa; 5 Boston University School of Medicine, 72 East Concord St, Boston, MA 02118 USA; 6 Harvard Center for Population and Development Studies, Cambridge, MA 02138 USA

**Keywords:** Psychology, Human behaviour, Risk factors

## Abstract

There are individual differences in health outcomes following exposure to childhood maltreatment, yet constant individual variance is often assumed in analyses. Among 286 Black, South African women, the association between childhood maltreatment and neurocognitive health, defined here as neurocognitive performance (NP), was first estimated assuming constant variance. Then, without assuming constant variance, we applied Goldstein’s method (Encyclopedia of statistics in behavioral science, Wiley, 2005) to model “complex level-1 variation” in NP as a function of childhood maltreatment. Mean performance in some tests of information processing speed (Digit-symbol, Stroop Word, and Stroop Color) lowered with increasing severity of childhood maltreatment, without evidence of significant individual variation. Conversely, we found significant individual variation by severity of childhood maltreatment in tests of information processing speed (Trail Making Test) and executive function (Color Trails 2 and Stroop Color-Word), in the absence of mean differences. Exploratory results suggest that the presence of individual-level heterogeneity in neurocognitive performance among women exposed to childhood maltreatment warrants further exploration. The methods presented here may be used in a person-centered framework to better understand vulnerability to the toxic neurocognitive effects of childhood maltreatment *at the individual level*, ultimately informing personalized prevention and treatment.

## Introduction

Only a proportion of individuals exposed to traumatic events develop adverse mental health conditions^1^, even though global surveys suggest that most people are exposed to life-threatening events^2^. There has been significant progress in understanding sources of this heterogeneity from the life-course perspective^3^. Additional progress has been made using approaches that estimate population heterogeneity, most notably by using latent growth mixture modelling (LGMM)^4–6^. The LGMM approach, which basically separates a general population of individuals into subgroups characterized by distinct patters of change over time, has been applied to a wide body of traumatic stressors ranging from wartime combat exposure^7^, job loss^8^, spousal and child bereavement^9^, campus shootings^10^, and natural disasters^11^. These studies have suggested that in part, heterogeneity in post-exposure outcomes can be explained by differences among individuals across domains as varied as cognitive style^12,13^, self-enhancement^14^, and subjective well-being^15^. Identifying these individual difference domains has clinical and public health implications because it can inform the early identification of individuals who might be vulnerable to adverse outcomes, ultimately informing prevention strategies to avert downstream adverse outcomes^16,17^.

While these population heterogeneity models provide insight into between-group differences in identified domains (e.g. the presence of social support^10^), the question of how *individuals* might be more or less vulnerable to adverse outcomes remains open. Specifically, individuals themselves within any single class derived by employing the LGMM approach might vary from each other in systematic and meaningful ways, yet this possibility is rarely directly interrogated because homoscedasticity (constant error variance) is assumed^18–21^. Approaches that do not make this assumption could potentially identify systematic variability, given that such variability may not be a random process^21^. Goldstein’s^22^ approach recognizes heteroscedasticity (non-constant error variance) and models “complex level-1 variation” as a function of a specified predictor. While the advantages of fitting models that relate to the amount of level-1 variability—or heteroscedasticity—have been highlighted in the methodological literature^23^, the substantive implications for understanding factors that systematically contribute to differential variation in health outcomes is not yet widely appreciated. The extant literature applying Goldstein’s^22^ methodology to understand individual variation in health outcomes has identified systematic heterogeneity in body mass index by low and middle income country residence^24^ and adult anthropometry by wealth and education^25^, suggesting non-random factors are driving some of the individual variation in these health indicators. These studies illustrate that understanding factors that systematically contribute to differential variation may have downstream clinical and public health implications, and may ultimately inform personalized clinical intervention and prevention strategies.

In the present study, we sought to apply Goldstein’s^22^ model of complex level-1 variation to neurocognitive performance as a function of exposure to childhood maltreatment among Black, South African women. There were two motivations for extending this model to a study of neurocognitive performance among these women. First, a robust literature documents an association between exposure to childhood trauma and alterations in brain systems including network architecture^26^ and structure^27^. Population-based studies have further demonstrated that exposure to childhood maltreatment is associated with impairment in academic functioning^28^ and environmental suppression of full scale IQ^29^. Given the implications of compromised neurocognitive competence on health and well-being across the lifespan associated with exposure to childhood maltreatment, we sought to quantify the magnitude of individual variability because understanding the factors that systematically contribute to differential variation in health outcomes might inform personalized approaches to prevention and treatment. Second, most extant research using statistical approaches that model heterogenous distributions of neurocognitive performance have generally relied on global north, White samples, with little diversity represented even though the adverse effects of structural determinants on health outcomes have been well documented^30^.

Given that this study was an exploratory analysis, we broadly hypothesized that exposure to childhood maltreatment would be associated with increased individual variability in neurocognitive performance compared to non-exposed individuals given prior findings documenting individual differences associated with exposure to childhood maltreatment^31,32^. We predicted that exposure to childhood maltreatment would be associated with increased variability in neurocognitive performance independent of the average association, even when controlling for other sources of potential variability including background demographic variables and psychiatric burden of depressive and posttraumatic stress symptoms.

## Methods

### Participants

Data were drawn from a prior study conducted to investigate the relationship between traumatic events, HIV infection, and behavioral and brain health among South African women^33,34^. To be included in the study, the participants had to be: (1) between the ages of 18 and 65, (2) able to read and write in either English and Afrikaans at the 5th grade level, and (3) healthy enough to undergo neuropsychological performance testing and magnetic resonance imaging (MRI) scans. The health-related conditions to merit exclusion were MRI contraindications including pregnancy, having taken psychotropic medications, being hepatitis positive, central nervous system infections or neoplasms, significant previous head injury, current seizure disorders, demonstrated cognitive impairment assessed on the International HIV Dementia Scale^35^ (HDS < 10), substance or alcohol abuse/dependence in the previous year assessed by clinical interview, a history of schizophrenia, bipolar disorder, or other psychotic disorders assessed by the Mini-International Neuropsychiatric Interview-Plus^36^.

### Procedure

From 2008 to 2015, potentially eligible women were recruited from hospitals, day clinics, and communities around Cape Town, South Africa by research assistants, research nurses, or with the help of physicians or counselors. Women who consented to participate were screened for eligibility by a phone or in-person interview. Those who met the initial eligibility criteria were invited to Stellenbosch University for screening by a physician, self-reported assessments, a neuropsychiatric interview, collection of a blood sample, and neuropsychological tests. The current study utilizes information collected by self-report measures, neuropsychological tests, and a blood sample. The neuropsychological tests were individually administered by a trained psychologist or a nurse in a private, quiet testing laboratory at a standardized time of day. The test administers followed a structured instruction manual to ensure consistency across the all tests.

The neuropsychological tests were conducted in English at the beginning of the data collection, and later in Xhosa when the translated instruments became available. The sample was balanced in testing language administration, and there were no systematic differences in sample characteristics between those who tested in English and those who tested in Xhosa^37^. Sociodemographic information, such as years of education and language spoken at home, was collected using self-reported assessments.

All participants provided written informed consent and were reimbursed for the transportation cost of ZAR250 to the data collection site. The primary study was approved by the ethics committee of Stellenbosch University (ethics reference number: N07/07/153), and all research was performed in accordance with relevant guidelines and regulations.

### Measures

#### Neurocognitive performance

A battery of 15 neuropsychological tests assessed performance across seven neurocognitive domains (see Table 1) selected on the basis of their sensitivity to trauma exposure^38,39^. These tests have also been widely utilized in international research settings^40,41^. Tests used in the current study were translated into Xhosa using standard adaptation techniques such as forward and backward translation, and modified as needed to fit the local cultural context using strategies have been successfully used in other African contexts^42^. Specifically, gemstones that appear in the verbal episodic memory test (HVLT) are unfamiliar in the local context, and were therefore replaced with vegetables. For the phonemic verbal fluency test, the original letters ‘F’ and ‘A’ were replaced by the new letters ‘I’ and ‘B’ for Xhosa speakers. Replacement letters were selected based on matching the rank ordered frequency in English and Xhosa dictionaries.

**Table 1:** Neuropsychological tests administered to all participants.

Domain	Test name	Abbreviation	Citation
Verbal fluency	Controlled Oral Word Association Test of phonemic fluency	COWAT	^66^
	Animal Test of semantic fluency	Animals	^67^
	Action Test of semantic fluency	Action	^67^
Executive function	Wisconsin Card Sorting Test–Computer version	WCST	^68^
	Color Trails 2	Color Trails 2	^69^
	Halstead Category Test–Computer version	Category	^70^
Information processing speed	Trail Making Test–A	TMT-A	^71^
	Wechsler Adult Intelligence Scale, Third Edition Digit-Symbol subtest	WAIS-III DS	^72^
	Wechsler Adult Intelligence Scale, Third Edition Symbol Search subtest	WAIS-III SS	^72^
	Stroop Word naming speed	Stroop Word	^73^
	Stroop Color naming speed	Stroop Color	^73^
	Color Trails 1	Color Trails 1	^70^
Verbal episodic memory	Hopkins Verbal Learning Test, Revised–total learning	HVLT-L	^74^
	Hopkins Verbal Learning Test, Revised–delayed recall	HVLT-R	^74^
Visual episodic memory	Brief Visuospatial Memory Test, Revised–total learning	BVMT-L	^75^
	Brief Visuospatial Memory Test, Revised–delayed recall	BVML-R	^75^
Attention/working memory	Paced Auditory Serial Addition Test	PASAT	^76^
	Wechsler Memory Scale-III Spatial Span Task	WMS-III SS	^72^

#### Childhood maltreatment

The Childhood Trauma Questionnaire-Short Form (CTQ-SF)^43^ is a retrospective self-report inventory with 28-items that assesses severity of exposure to different types of childhood trauma. The items were introduced with the statement, “These questions ask about some of your experiences growing up as a child and a teenager. For each question, circle the number that best describes how you feel”. Each item score ranges from 1 (“never true”) to 5 (“very often true”), producing scores of 5–25 for each subscale. The five subscales are stratified by emotional abuse, physical abuse, sexual abuse, emotional neglect, and physical neglect. Some items are reverse coded so that a higher score reflects a more severe exposure to maltreatment. The instrument demonstrated high internal consistency (Cronbach’s $\alpha$ = 0.85). The sum score was used as a continuous measure in all analyses.

#### Mental health symptoms

The Center for Epidemiologic Studies Depression Scale (CES-D)^44^ is a 20-item self-report measure commonly used to screen for symptoms of depression experienced in the previous week. Item values are summed for a possible range from 0 to 60, with higher total scores indicating increasing severity. Traumatic stress symptoms were assessed using the Davidson Trauma Scale (DTS)^45^, which is a 17-item, self-rated questionnaire assessing posttraumatic stress disorder symptoms corresponding to the DSM-IV^46^ symptom criteria of PTSD. Total scores are generated by summing ratings of both frequency and severity of target symptoms, with higher scores corresponding to greater symptom burden.

### Covariates

All analyses were adjusted for age (continuous), education level (less than or equal to grade 8 vs. greater than grade 8), household income (less than ZAR10,000 vs. higher), employment status (yes vs. no), marital status (single vs. married/cohabitating vs. separated/divorced/widowed), HIV status (positive vs. negative), depression symptoms (continuous), and traumatic stress symptoms (continuous). Education levels and household income were adjusted as binary variables as indicated above because the distributions were highly skewed.

### Analytic approach

To assess whether variability in neurocognitive performance (NP) varied with severity of exposure to childhood trauma, we constructed two types of linear models, one assuming homogeneous variance [Ordinary Least Squares (OLS); Model 1] and the other assuming heterogeneous variance (complex level-1; Model 2). For the first OLS models, we specified a linear regression with the conventional homogeneous variance assumption, or homoscedasticity, adjusting for all pre-specified covariates (age, education level, household income, employment status, marital status, HIV status, depression and traumatic stress symptoms). Then, following Goldstein’s method^22^ to estimate complex level-1 variation (Model 2), we relax this commonly violated assumption by modelling the variance of neurocognitive performance as a function of exposure to childhood maltreatment. Here, the variance in neurocognitive performance (${\upsigma }_{{\mathrm{e}}_{0}}^{2}$) is described as $e \sim N\left(0, {\upsigma }_{{\mathrm{e}}_{0}}^{2}\right)$. By summarizing the residual variance as a single estimate, the conventional homoscedasticity assumption states that the variance ${\upsigma }_{\mathrm{e}}^{2}$ is constant across all types of individuals. In Model 2, The neurocognitive performance variance is now described as a variance–covariance matrix $$\left[ {\begin{array}{*{20}c} {e_{0} } \\ {e_{1} } \\ \end{array} } \right]\sim N\left( {0,\left[ {\begin{array}{*{20}c} {\sigma _{{{\text{e}_0} }}^{2} } & - \\ {\sigma _{{e_{0} e_{1} }} } & {\sigma _{{e_{1} }}^{2} } \\ \end{array} } \right]} \right)$$ where ${e}_{0}$ and ${\upsigma }_{{\mathrm{e}}_{0}}^{2}$ are the residuals for those who scored zero on trauma exposure and their variance, respectively. The covariance ${\sigma }_{{e}_{0}{e}_{1}}$ and variance ${\upsigma }_{{e}_{1}}^{2}$ can be understood as linear and quadratic parts of the variance function. The variance function for each value of trauma exposure is estimated by ${\upsigma }_{{\mathrm{e}}_{0}}^{2}+2{\sigma }_{{e}_{0}{e}_{1}}\times {x}_{1}+{\upsigma }_{{e}_{1}}^{2}\times {x}_{1}^{2}$ where ${x}_{1}$ is a continuous trauma exposure variable. That is, the neurocognitive performance variance is modelled as a quadratic function reflective of the level of childhood maltreatment exposure. To visualize how average neurocognitive performance and the variability simultaneously change with the level of trauma exposure performance, we provide graphs with the predicted values of neurocognitive performance by trauma exposure accounting for all other covariates and their 95% variation bounds (the lower and upper bounds wherein 95% of the observations lie) calculated by average neurocognitive performance (NP) $\pm 1.96\times \sqrt{Var(NP)}$ (see also Lee^47^ for further explanation). Lastly, we conducted likelihood ratio tests (LRT) comparing Model 1 and Model 2 to see if heterogeneity of the variance is statistically significant. For all models testing for mean differences, we set our *p* value cutoff at the traditional < 0.05 level. Then, we set the *p* value cutoff to < 0.10 for variance estimates following convention previously recommended given that the null hypothesis is at the boundary of the parameter space^48^. All analyses were performed using R2MLwiN package^49^ that calls MLwiN 3.04^50^ within R (R Core Team, 2020)^51^.

## Results

The analytic sample included 286 participants. The mean age was 30.62 (SD 7.83, range 18–50). The majority were Black (98.3%) and spoke Xhosa at home (94.8%). Most participants had some high school education with no diploma (87.4%) and reported low combined annual household income (< ZAR10,000 or $781USD), which is far below the South African 2017 average household net-adjusted disposable income of $10,872 USD. The sample included 25.9% of those who were married or cohabitating, 3.8% of the separated, divorced, or widowed, and 70.3% single women. Some were the primary breadwinner of their households (31.8%) or employed (28.3%). They had, on average, 1.57 children (SD 1.24). The mean CES-D score was 11.75 (SD 14.84), the mean DTS score was 17.40 (SD 30.52), and about half were HIV-positive (48.6%). 30.42% of the sample scored above the typically used clinical cutoff of ≥ 16 on the CESD, and 18.18% of the sample scored above the recommended clinical cutoff value of ≥ 40 for the DTS.

There were some significant differences in mean CTQ score by some demographic characteristics (Table 2). Women with a Grade 8 or less education level had significantly higher CTQ scores (M 56.90, SD 22.6) compared to those women who had greater than Grade 8 level of education [M 45.70, SD 18.8, *F*(2, 284) = 10.66, *p* = 0.001]. CTQ scores also varied by HIV status such that women with positive HIV status had a higher mean CTQ score (M 54.70, SD 20.30) compared to women with negative HIV status [M 39.50, SD 15.6, *F*(2, 284) = 56.05, *p* < 0.001].

**Table 2:** Childhood Trauma Questionnaire (CTQ) sum scores, subscale scores, and comparison of CTQ sum scores by selected sociodemographic indicators, N = 286.

	CTQ mean (SD)	*n*	*%*	*F*	*p*	*Min*	*Max*
Education level*						5	14
Grade 8 or less	56.9 (22.6)	31	10.8	10.660	0.001		
Higher than Grade 8	45.7 (18.8)	255	89.2				
**Household annual income**						N/A^t^	N/A
Less than ZAR10,000	47.7 (19.7)	248	86.7	3.159	0.077		
Greater than ZAR10,000	41.5 (18.0)	38	13.3				
**Marital status**							
Married/cohabitating	48.1 (16.0)	74	25.9	1.779	0.171		
Separated/divorced/widowed	43.0 (16.4)	11	3.8				
Single	48.2 (20.7)	201	70.3				
**Employed**							
Yes	47.1 (18.1)	81	28.3	0.073	0.787		
No	46.8 (18.1)	205	71.7				
**HIV status**							
Positive	54.7 (20.3)	139	48.6	56.05	< 0.001		
Negative	39.5 (15.6)	147	51.4				
**CTQ total**	46.6 (19.0)					25	114
Physical neglect	9.5 (4.3)					5	24
Emotional neglect	11.0 (5.5)					5	25
Emotional abuse	10.7 (5.8)					5	25
Physical abuse	8.5 (5.3)					5	25
Sexual abuse	7.0 (4.4)					5	25

*Education level was measured as years of school completed; ^a^nnual income was measured as a binary variable therefore min and max values are not available.

Next, we examined CTQ scores by subscale and clinical cutoffs as established by Bernstein^43^ to characterize this cohort in terms of specific abuse and neglect experiences (see Table 3). The overall mean score on the CTQ was 46.61 (SD 19.0), with a minimum score of 25 to a maximum of 114 (see Table 2). Subscales had a minimum and maximum range of 5–25. Emotional neglect had the highest value with a mean of 10.7 (SD 5.8), followed by emotional abuse (M 10.7, SD 5.8), physical neglect (M 9.5, SD 4.3), physical abuse (M 8.5, SD 5.3), and sexual abuse (M 7.0, SD 4.4).

**Table 3:** Severity level of CTQ abuse and neglect subscales stratified by frequency and percent of sample scoring in each respective severity range (n = 286).

Severity	EA	PA	SA	EN	PN
	n (%)	n (%)	n (%)	n (%)	n (%)
None or minimal	128 (44.8)	179 (62.6)	204 (71.3)	117 (40.9)	117 (40.9)
Low to moderate	64 (22.4)	31 (10.8)	25 (8.7)	68 (23.8)	62 (21.7)
Moderate to severe	29 (10.1)	22 (7.7)	27 (9.4)	41 (14.3)	37 (12.9)
Severe to extreme	65 (22.7)	54 (18.9)	30 (10.5)	60 (21.0)	70 (24.5)

Category value ranges are defined following Bernstein^43^.

*EA* CTQ emotional abuse, *PA* CTQ physical abuse, *SA* CTQ sexual abuse, *EN* CTQ emotional neglect, *PN* CTQ physical neglect.

Examination of ordinary least squares (OLS) model coefficients show consistent negative associations across three specific tests of information processing speed and higher CTQ scores (see Table 4). Adjusting for all covariates including age, education, HIV status, marital status, employment status, income, depression and PTSD symptoms, women with higher CTQ scores, on average, had lower scores on the WAIS Digit Symbol task (unadjusted $\beta$=− 0.13, 95% CI [− 0.022, − 0.04], *p* = 0.004), Stroop Word task (unadjusted $\beta$=− 0.17, 95% CI [− 0.028, − 0.05], *p* = 0.004), and the Stroop Color task (unadjusted $\beta$=− 0.14, 95% CI [− 0.024, − 0.05], *p* = 0.003). No other NP tests showed significant associations by severity of exposure to childhood maltreatment.

**Table 4:** Coefficients for the constant variance [$\beta$ (95% confidence interval)] and complex level-1 models [${\sigma }^{2}$ (95% confidence interval] of exposure to childhood trauma as assessed by continuous score on the CTQ (Childhood Trauma Questionnaire) regressed on individual tests of neurocognitive performance, controlling for covariates.

	Homogeneous variance		Heterogeneous variance			LRT *p*
	$\beta$(95% CI)	*p*	${\sigma }^{2}$ (95% CI) (intercept)	Covariance	${\sigma }^{2}$ (95% CI) (exposure)	*p*
**Verbal episodic memory**						
HVLT-L	− 0.01 (− 0.03, 0.02)	0.783	17.06 (13.83, 20.28)	0.05 (− 0.03, 0.14)	− 0.01 (− 0.01, 0.01)	0.604
HVLT-DR	− 0.01 (− 0.02, 0.01)	0.351	4.44 (3.49, 5.39)	0.01 (− 0.01, 0.03)	− 0.01 (− 0.01, 0.01)	0.740
**Visual episodic memory**						
BVMT-L	0.01 (− 0.05, 0.05)	0.966	42.36 (31.78, 52.96)	− 0.05 (− 0.35, 0.24)	0.02 (− 0.01, 0.05)	0.250
BVMT-DR	− 0.01 (− 0.03, 0.02)	0.639	7.57 (5.71, 9.43)	− 0.01 (− 0.05, 0.05)	0.01 (− 0.01, 0.01)	0.393
**Information processing speed**						
WAIS DS	**− 0.13 (− 0.23, − 0.04)**	**0.004**	134.59 (102.61, 166.56)	− 0.25 (− 1.12, 0.63)	0.04 (− 0.03, 0.12)	0.261
WAIS SS	− 0.02 (− 0.06, 0.04)	0.711	50.21 (39.18, 61.26)	− 0.11 (− 0.42, 0.19)	0.01 (− 0.02, 0.03)	0.701
TMT-A	0.14 (− 0.03, 0.30)	0.110	**525.16 (407.49, 642.83)**	**3.38 (0.64, 6.12)**	**− 0.08 (− 0.30, 0.133)**	**0.063**
Color Trails 1	− 0.09 (− 0.24, 0.07)	0.275	527.35 (422.92, 631.77)	0.70 (− 2.07, 3.47)	− 0.08 (− 0.23, 0.08)	0.739
Stroop Word	**− 0.18 (− 0.30, − 0.05)**	**0.004**	266.26 (206.69, 325.84)	− 0.05 (− 1.63, 1.53)	0.02 (− 0.10, 0.14)	0.942
Stroop Color	**− 0.14 (− 0.24, − 0.05)**	**0.003**	156.15 (119.13, 193.17)	0.08 (− 0.90, 1.05)	0.03 (− 0.05, 0.18)	0.419
**Executive functions**						
Color Trails 2	0.17 (− 0.17, 0.51)	0.329	**2001.95 (1666.32, 2337.58)**	**13.36 (3.19, 23.53)**	n/a*	**0.005**
WCST-64	− 0.03 (− 0.13, 0.06)	0.493	123.57 (93.29, 153.85)	− 0.71 (− 1.61, 0.18)	0.08 (0.01, 0.16)	0.262
Category	0.01 (− 0.15, 0.17)	0.922	665.72 (540.63, 790.81)	1.65 (− 1.66, 4.97)	− 0.15 (− 0.31, − 0.01)	0.274
Stroop-CW	− 0.04 (− 0.12, 0.03)	0.260	**84.51 (63.22, 105.81)**	**0.34 (− 0.20, 0.88)**	**0.03 (− 0.02, 0.08)**	**0.049**
**Verbal fluency**						
COWAT	0.01 (− 0.06, 0.07)	0.911	79.38 (62.41, 96.36)	− 0.23 (− 0.71, 0.24)	0.01 (− 0.02, 0.04)	0.594
Animals	− 0.01 (− 0.04, 0.01)	0.183	8.29 (6.46, 10.13)	− 0.02 (− 0.07, 0.03)	0.01 (− 0.01, 0.01)	0.745
Action	− 0.01 (− 0.03, 0.03)	0.989	14.09 (11.18, 17.00)	0.03 (− 0.04, 0.11)	− 0.01 (− 0.01, 0.01)	0.741
**Working memory**						
PASAT 50	0.01 (− 0.05, 0.07)	0.795	80.39 (63.95, 96.83)	− 0.51 (− 0.99, − 0.02)	0.01 (− 0.02, 0.04)	0.126
WMS-III SS	0.01 (− 0.02, 0.02)	0.824	10.00 (7.91, 12.09)	0.05 (0.01, 0.10)	− 0.01 (− 0.01, − 0.01)	0.184

Log-likelihood ratio test (LRT) values comparing the variance estimates from OLS models with complex level-1 models are reported in the last column.

All models adjusted for age, education level, HIV status, marital status, employment status, income, depression and PTSD symptoms levels.

Mean and variance estimates that lie between − 0.01 and 0.01 were rounded to the closer of the either values. The rounding does not influence the inference as these estimates were not noticeably different from zero.

Some variance estimates were allowed to be negative, which is intuitively confusing. However, these estimates should be interpreted as part of a variance function, which is non-negative.

*LRT* loglikelihood ratio test, *WCST* Wisconsin Card Sorting Test, *Stroop-CW* Stroop Color-Word Incongruence, *TMT-A* Trail Making Test, Version A, *WAIS*-*III DS* WAIS-III Digit Symbol, *WAIS-III S–S* WAIS-III Symbol Search, *HVLT-R*, *L* Hopkins Verbal Learning Test, Revised, Learning, *HVLT-R, DR* Hopkins Verbal Learning Test, Revised, Delayed Recall, *BVMT-R, L* Brief Visuospatial Memory Test, Revised, Learning, *BVMT-R, DR* Brief Visuospatial Memory Test, Revised, Delayed Recall, *PASAT* Paced Auditory Serial Addition Task, *WMS-III SS* WMS-III Spatial Span.

*n/a is entered here because the quadratic function did not converge. Therefore we ran a linear function.

The last four columns of Table 4 demonstrate differential variation in neurocognitive performance by CTQ score across three NP tests at the *p* < 0.10 level, as evidenced by log likelihood ratio tests comparing OLS and complex model parameters across NP tests in domains of executive function (Color Trails 2; *Χ*^2^ (df = 1) = 4.40, *p* = 0.036) and Stroop Color-Word; *Χ*^2^ (df = 2) = 6.19, *p* = 0.045), and information processing speed (TMT-A; *Χ*^2^ (df = 2) = 5.87, *p* = 0.053) Together, results indicate significant individual variation in neurocognitive performance, or heteroskedasticity, relative to increased exposure to childhood maltreatment. Importantly, across these three tests, *mean differences* in neurocognitive performance did not vary by CTQ score. To illustrate we compare residual variance from a constant variance model (OLS) and residual variance from the complex level-1 model by calculating var(intercept) + 2 × cov + var(exposure) × exposure. Thus, the range of residual variance is calculated based on the minimum and maximum CTQ score used to derive the modelled min and max residual variance. Results showed that while residual variance in TMT-A was estimated as 497.31 in the constant variance (OLS) Model 1, the minimum and maximum residual variance from the complex variance model ranged from 345.53 to 662.80 by CTQ score. Similarly, residual variance in the Color Trails 2 task was estimated as 1992.41 in the OLS model, but actually ranged from 1438.04 to 3816.36 by CTQ score. Finally, residual variance in the Stroop–Color Word test was estimated as 94.96 in the OLS model, but actually ranged from 80.72 to 268.49 by CTQ score.

**Figure 1:**
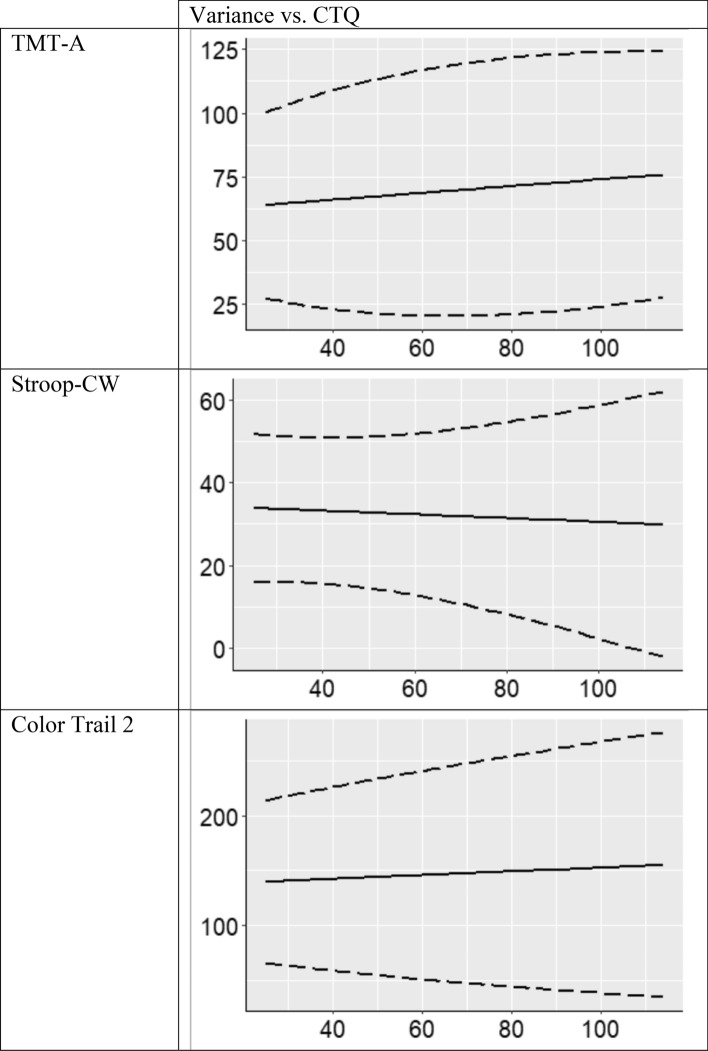
Predicted values of neurocognitive performance by CTQ score derived from complex level-1 models demonstrating statistically significant patterns of individual-level heterogeneity. Dotted lines delineate the upper and lower bounds wherein 95% of observed neurocognitive performance scores by CTQ level lie ($\pm 1.96\times \sqrt{\mathrm{Var}(\mathrm{NP})}$).

Finally, to visualize how average neurocognitive performance and individual variability simultaneously change with the level of exposure to childhood maltreatment, we provide graphs in Fig. 1 with the predicted values of neurocognitive performance by trauma exposure accounting for all other covariates and its 95% variation bounds calculated by average neurocognitive performance ($\pm 1.96\times \sqrt{\mathrm{Var}(\mathrm{NP})}$). These graphs demonstrate statistically significant patterns of individual heterogeneity at the *p* < 0.10 level, including increased NP variability by maltreatment exposure in tests of executive function (Stroop Color-Word and Color Trails 2), and lower variability in a test of information processing speed (TMT-A).

## Discussion

In this exploratory analysis of neurocognitive performance (NP) among Black, South African women, we find evidence to suggest systematic individual variation in some NP tests by exposure to self-reported childhood maltreatment. First, constant variance OLS models identified a significant association of lower scores in three tests of information processing speed (Digit-symbol test, Stroop Word, and Stroop Color) with increasing exposure to childhood maltreatment, meaning that exposure to maltreatment *on average* was associated with worsened performance in these tests without evidence of affecting individual variability. On the other hand, when individual heterogeneity was modelled following Goldstein’s^22^ complex level-1 approach (Model 2), we found significantly greater variability on tests of executive function (Stroop Color-Word and Color Trails 2) and lower variability in a test of information processing speed (TMT-A) with increasing level of maltreatment exposure. Notably, models assuming constant variance did not demonstrate a significant *average* effect of childhood maltreatment exposure in these same three tests. Taken together, results suggest that even in the absence of an overall correlation with CTQ, complex level-1 models detect significant individual variability (i.e. within-population) in some tests of NP performance. This implies the presence of systematic factors (beyond the demographic and psychological variables controlled for in the present study) that may impact the association between executive functioning and information processing speed among maltreatment exposed individuals compared to non-maltreated individuals. To better understand this pattern, subsequent stratified analyses by meaningfully defined subgroups with potentially different sets of risk factors relevant for each are necessary.

Our results are consistent with prior work documenting associations among exposure to childhood maltreatment and altered neurocognitive performance. For example, our findings regarding mean differences from OLS models are consistent with prior work documenting the association between slowed processing speed and exposure to childhood maltreatment^52^. However, when we relax the assumption that individual variation in NP performance by childhood maltreatment exposure is constant, we indeed find evidence of underlying systematic individual variation in NP by childhood maltreatment. The specific domains wherein significant individual variation was detected overlap with prior work implicating these functions in post-trauma exposure functioning, including executive functions^53,54^ and attention^54,55^. Our exploratory findings augment this literature by demonstrating the additional presence of individual variability, implying that existing literature on the relationship between trauma exposure and neurocognition should be interpreted with the understanding that in addition to average group differences, additional analyses modelling individual variability may augment investigations into factors associated with systematic patterning at the individual level.

Analytically, our findings noting differential variability in NP by childhood maltreatment have two explanations. First, the *same* sets of factors may effect NP in exposed vs. non-exposed groups, but the magnitude of that effect varies by the severity of exposure to childhood maltreatment. Such an interaction effect, if found, could help identify specific brain-based functions that are particularly susceptible to adverse childhood experiences. The second explanation is that *different* sets of factors affect NP performance in the exposed vs. non-exposed groups. That is, exposure to childhood maltreatment initiates a cascade of developmental consequences that are quantitatively different than those experienced by those not exposed. Prior findings implicating sensitive periods^56^, altered social functioning^57^, and cognitive processing^58^ for example, could provide a basis for testing further hypotheses regarding specific factors that drive individual variability in post-exposure functioning.

Descriptively, our results suggest that individual performance in tests of executive function and information processing speed is characterized by systematic variation relative to exposure to childhood maltreatment. The next step for future research is to address the question as to why variability might be different among exposed and unexposed individuals. Descriptively, increasing variance can be interpreted as a marker of vulnerability. Yet within that, why some individuals evidence an association with decrements in neurocognitive performance, while others appear robust to adverse effects, remains open. It may be that factors known to moderate stress outcomes such as social support^10^, educational attainment^59^, and neighborhood assets^60^ act at the individual level to increase, or reduce, risk for compromised NP. It could also be that specific types of maltreatment exposure (e.g. physical vs. sexual abuse) are associated with different patterns of individual level NP variability, a possibility that the present study was underpowered to examine but a potentially fruitful line of future research consistent with a developmental perspective^61,62^. Future research can directly interrogate this possibility by stratifying samples by exposed and unexposed at specific developmental periods, and by specific types of maltreatment, and assess the association between health outcomes and candidate buffering factors in those neurocognitive domains specifically demonstrating increased individual variability.

Interestingly, our results also suggest that exposure to childhood maltreatment is associated with reduced variability in a test of information processing speed. Though difficult to interpret and highly speculative, reduced dispersion might suggest the possibility of compensatory processes. For example, prior work has found evidence of reduced nodal connectivity in brain network architecture among individuals resistant to psychiatric burden in the aftermath of exposure to childhood trauma exposure^26^. Future work can directly test this hypothesis by examining functioning and health outcomes among individuals with exposure to childhood trauma as a function of performance in the specific neurocognitive domains shown to have reduced variability at the individual level. Alternatively, an elevated CTQ sum score could reflect exposure to multiple subtypes of maltreatment, and reduced variability in information processing speed is consistent with equifinality in that different types of adversity may eventuate similar outcomes across information processing speed functions^63^. To further test this possibility, better powered samples would be needed to stratify models by CTQ subscale.

Several limitations should be taken into account when interpreting results of this exploratory study. First, childhood maltreatment was ascertained using a self-report measure. Though a commonly used ‘gold-standard’ measure, there is the possibility that reporting of childhood maltreatment was subject to recall bias or subjective affective state^64^. Though we did control for symptoms of depression and PTSD as potential sources of affective bias, we cannot rule out the possibility that unmeasured factors influenced disclosure of childhood maltreatment. A related limitation is that the timing of childhood maltreatment exposure was not assessed. Therefore, we do not know how much time passed since the exposure event, or the developmental period in which the exposure occurred, which may vary considerably among individuals in the study. Though study participants were generally young (M 28.85, SD 8.97, range 18–54), this limitation should still be taken into consideration when interpreting findings. A related limitation pertains to the cross-sectional nature of our dataset wherein the direction of association between childhood maltreatment and downstream cognitive deficits cannot be determined^65^. Without prospective data, we are unable to ascertain level of cognitive functionating prior to exposure to maltreatment; it could be that individuals with greater baseline individual variability are more likely to experience exposure to childhood maltreatment. The fourth important limitation is that our sample was relatively small compared to prior studies^24,25^ applying this method, and we may have been underpowered to detect effects, especially in subtypes of maltreatment exposure. Future studies with prospective data on larger samples are needed to extend this work. A final related limitation to the study is the potential inflation of significance in light of the effects of multiple testing. We ran several similar models across 15 specific tests of neurocognitive function. We suggest risk of Type-1 error is slightly mitigated by the fact that NP tests were significantly different from one another in method, domain assessed, and administration. However, we were underpowered to introduce Bonferroni corrections for multiple testing, and future analyses should be conducted on larger sample sizes.

Modelling individual variability neurocognitive performance by exposure to childhood maltreatment has two important implications. First, assuming constant variance may obstruct the capacity to meaningfully ascertain the presence of individual heterogeneity in neurocognitive functioning associated with trauma exposure. That is, some individuals might be at more risk for compromised neurocognitive performance compared to others, but this would be impossible to detect when comparing group means across exposed and unexposed individuals. Second, meaningful decomposition of hypothesized variability might inform our understanding of individual vulnerability to the toxic neurocognitive effects of childhood maltreatment. That is, modelling individual variation directly could detect meaningful systematic patterning of individual differences, pointing towards early identification of vulnerable individuals to tailor prevention and treatment. An important line of future person-centered research^61^ could be employed by segmenting exposed individuals by the subtype of maltreatment and severity to help interpret patterns of systemic individual variability. Understanding sources of heteroskedasticity could likely provide greater insight into the factors that systematically contribute to differential variation in neurocognitive functioning associated with trauma exposure, with significant implications for more tailored and targeted interventions once vulnerable individuals are identified. Such future investigations can also go further in providing empirical evidence to better understand the factors that are likely to drive this individual variability, such as those previously mentioned including social support, educational attainment, and neighborhood assets, for example. Then, when adequate sample sizes are available, future research may also employ genome wide association approaches to investigate the combined impact of genetic variants, environmental exposure, and psychosocial factors on neurocognitive performance by maltreatment exposure. In conclusion, our study results suggest that analyses considering systematic patterning of both means and variances in tandem may significantly augment our knowledge base, and potentially identify factors that can inform individualized treatment and prevention.
